# Exploring Pharmacy Technician Roles in the Implementation of an Appointment-Based Medication Synchronization Program

**DOI:** 10.3390/pharmacy8010028

**Published:** 2020-03-03

**Authors:** Chelsea Renfro, Davis Coulter, Lan Ly, Cindy Fisher, Lindsay Cardosi, Mike Wasson, Kenneth C. Hohmeier

**Affiliations:** 1 Department of Clinical Pharmacy and Translational Science, University of Tennessee Health Science Center College of Pharmacy, Memphis, TN 38163, USA; crenfro@uthsc.edu (C.R.); dcoulter@uthsc.edu (D.C.); lly2@uthsc.edu (L.L.); 2 Kroger Pharmacy, Memphis, TN 38103, USA; cindy.fisher@kroger.com (C.F.); mike.wasson@kroger.com (M.W.); 3 Methodist South Bedside Delivery Program, Memphis, TN 38116, USA; linbblan@uthsc.edu; 4 Department of Clinical Pharmacy and Translational Science, University of Tennessee Health Science Center College of Pharmacy, Nashville, TN 37211, USA

**Keywords:** medication synchronization, service implementation, community pharmacy, pharmacy technicians

## Abstract

The objective of this study was to qualitatively explore the role of pharmacy technicians in the implementation of an appointment-based model (ABM) medication synchronization program. The purposeful sampling of technicians working within six different locations of a supermarket chain pharmacy in Mississippi and Tennessee was carried out, and the technicians were interviewed between January and April 2018. A semi-structured interview guide was developed based on the Consolidated Framework for Implementation Research (CFIR). Questions gathered information around pharmacy technician demographics and CFIR domains (process, inner setting, outer setting and intervention characteristics). Interviews were audiotaped and transcribed. Two members of the research team performed thematic content analysis. Six full-time, certified pharmacy technicians with 8.3 ± 2.7 years of experience were interviewed. Findings suggest that including hands-on experience with program software is needed during training to successfully implement ABM. A barrier to implementation was the time needed to complete ABM tasks as compared to other tasks. Although some barriers exist regarding implementation, technicians believe that overall, this program has positive benefits for patients. Results from this study signify that ABM implementation can be challenging. Better ABM portal integration with the pharmacy patient profile and appropriate workforce budgeting are key to continued success.

## 1. Introduction

According to the Centers for Disease Control and Prevention (CDC), one in four adults suffer from at least two or more chronic diseases such as diabetes, dyslipidemia, and hypertension [1]. The World Health Organization (WHO) found that only 50% of patients, on average, in developed countries with chronic diseases are adherent to their medications [2]. Medication nonadherence in patients with chronic conditions escalates direct health care costs nearly $100–$300 billion dollars each year [3]. Medication synchronization is a program proven to increase adherence, reduce emergency department visits and reduce hospitalizations for these patients [4,5]. Community pharmacies have incorporated medication synchronization into their workflow to improve quality of care and medication adherence. Medication synchronization is the alignment of a patient’s medication refills to a single date each month. Other features can be added in conjunction, such as comprehensive medication reviews (CMRs) and delivery [6,7].

The Appointment Based Model (ABM), one type of medication synchronization, is a patient care model where patients have one or two appointed days per month to pick up all medications [7]. The pharmacist performs additional patient care services, such as a comprehensive medication review (CMR), on that day to evaluate therapy and answer any questions or concerns from the patient [8]. Approximately 20,000 community pharmacies have implemented this service in the United States, and it is predicted to expand [9]. The medication synchronization component of ABM may be implemented by personnel at the patient’s pharmacy, or via a call center that identifies appropriate patients to enroll and places medication orders into pharmacy workflow. These programs allow pharmacies to clarify medication regimens, for stakeholders to enable optimization of medications while improving predictability of workflow and workload [6,7,8,9].

Successful process implementation requires buy-in from all members of the healthcare team [10]. Given the demands on pharmacist time, new service implementation in community pharmacy is frequently met with barriers [11,12,13]. While pharmacists deliver the clinical components of ABM, pharmacy technicians have a vital role in the implementation process for this service. The exploration of the pharmacy technician perspectives is crucial to understand how to overcome the hurdles facing ABM and medication adherence. There is limited research on roles, responsibilities, and challenges faced by pharmacy technicians in ABM implementation. The objective of this study was to qualitatively explore the role of pharmacy technicians in the implementation of an appointment-based medication synchronization program.

## 2. Materials and Methods 

### 2.1. Recruitment and Participants

Pharmacy technicians working one regional division of a large community pharmacy chain in either Mississippi or Tennessee were recruited. A purposeful sampling approach was used to recruit participants, whereby key informants were selected based on their exposure to ABM implementation, rather than selected randomly. The researchers were provided with a list of pharmacies from which subjects could be contacted, and researchers subsequently contacted and consented participants via telephone. Participants were stratified based on their pharmacy’s type of ABM used (in-store technician call model or an off-site call center model) and ABM performance (as defined by internal pharmacy measures of ABM implementation) within the supermarket chain pharmacy’s division. Participants had no prior experience with ABM prior to the study. This study was approved by the Institutional Review Board at the researchers’ university. All subjects gave their informed consent for inclusion before they participated in the study. The study was conducted in accordance with the Declaration of Helsinki, and the protocol was approved by the Ethics Committee of the University of Tennessee Health Science Center (18-05758-XM).

### 2.2. Data Collection

One member of the research team, with training in qualitative research, conducted 6 in-depth, semi-structured interviews from January to April 2018. Interviews lasted approximately 60 minutes and were conducted either via telephone or in person at the workplace. To best understand individual technician perspectives about ABM implementation, semi-structured interviews were chosen as compared to other methods, such as focus groups, which have the potential to obtain a consensus. [14].

A semi-structured interview guide was developed (Table 1) based on the Consolidated Framework for Implementation Research (CFIR) [10]. The interview guide was pilot tested with technicians at the same supermarket chain in a different division. CFIR consists of 37 constructs developed to synthesize a unified typology of implementation and dissemination theories and frameworks. The interview guide included questions categorized within four CFIR domains: (1) process (i.e., champion, engaging, innovation participants; (2) inner setting (i.e., relative priority, readiness for implementation, access to knowledge and information; (3) outer setting (i.e., needs and resources of those served by the organization); and (4) intervention characteristics (i.e., adaptability). A verbal consent statement was gathered prior to conducting the interview. The interviews were audiotaped and transcribed in their original format. Field notes were made during the interviews and added to the transcripts. 

### 2.3. Data Analysis

Using the CFIR codebook, two members of the research team (CR and DC) analyzed thematic content [10]. The initial session consisted of both researchers identifying preliminary codes and subthemes and resolving differences through active discussion. Afterwards, the researchers independently translated the remaining transcripts and met for a second session to identify any further emerging codes or subthemes that surfaced. Transcripts were analyzed using NVivo12 (QSR International Pty Ltd., 2018). Consistent themes observed were mapped to the constructs of the CFIR. The Consolidated Criteria for Reporting Qualitative Studies checklist was used to guide the reporting of qualitative methods and findings [15].

## 3. Results

A total of six participants were interviewed. Recruitment was stopped after six technicians since theme saturation (i.e., occurrence of similar themes with no new information collected) was reached [16]. Participants were full-time, certified pharmacy technicians with 8.3 ± 2.7 years of experience. Participants worked an average of 39.5 ± 1.1 hours per week. All participants interviewed had been employed at the supermarket chain studied for their entire career. 

### 3.1. Inner Setting of ABM Implementation 

Questions exploring the Access to Knowledge and Information construct found that while training effectively described the utility of the program, technicians were not always familiarized with the ABM support software adequately (Table 2). One participant expressed that the overall training was "useless” due to the lack of specific ABM user interface training. Technicians preferred the hands-on instruction. Technician training consisted of a presentation by a pharmacist or shadowing a pharmacist during the enrollment process for one training session. Repeat sessions were available if needed. 

Relative Priority of the program was a barrier because participants often felt they did not have enough time allocated to complete this task. Other time-consuming duties and initiatives in the pharmacy technicians stressed the already limited workforce hours allocated. This program was of secondary importance to the core operations of the pharmacy due to time constraints.

### 3.2. Outer Setting of the ABM Implementation Environment

The Patient Needs and Resources construct exploration in the technician interviews found that technicians perceived that patients benefit from the simplification of their medication regimens and the convenience of having to pick up once per month or once per quarter. Notably, adherent patients on a stable regimen were said to benefit the most from the program. The convenience of ABM was reported to allow patients to make fewer trips to the pharmacy. Technicians reported that some elderly patients had an aversion to change and a desired to remain on their current schedule of medication orders. Technicians perceived patient concerns about the increased single, monthly cost per pharmacy visit, as the cost of their monthly medication regimen would no longer be spread out over the entire month.

### 3.3. Intervention Characteristics Enabling ABM Success

The inability to adapt the ABM program to local needs was described as a barrier by technicians. The lack of integration of the ABM platform with the pharmacy dispensing software system caused prescriptions to “fall off” or old prescriptions to be filled. There was no dynamic update to accurately reflect the patient profile in the ABM portal, so technicians reported frustration managing medications manually (calculate the days’ supply on hand and fill the corresponding, appropriate quantity) to fit the ABM profile. The lack of ABM integration to the patient profile was identified as a critical issue with the program by pharmacy team members, that led to disrupted workflow and unneeded fills. The listing of every medication a patient has had on the ABM profile was described as a “busy” distraction that was a source of confusion when technicians are attempting to enroll patients with multiple medications.

### 3.4. Process ABM Follows for Success 

For the construct of champion, having a technician responsible for leading ABMS implementation and working with the team to achieve ABM goals was perceived to yield successful goal achievement and increased pharmacy staff buy-in. Roles reported included; taking the initiative to engage patients on the ABM enrollment list and managing the existing patient fills. These champions would actively listen to patients and suggest ABM to those individuals who expressed that travel to the pharmacy or complexity of regimen was a concern for them. Teams with a champion reported an increase in successful enrollments. 

Enrollment of Innovation Participants happened one of three ways: a patient would ask to have medications synchronized for convenience’s sake, a technician would identify the need based upon a past relationship with a patient, or the ABM program or sync center would (central location with telephonically available pharmacy technicians trained in medication synchronization) generate a list of patients to enroll. Here was an identified need for better communication from the sync center to the pharmacy, so that the correct patients could be identified. The ABM program is structured to automatically generate appropriate patients, but technicians reported making judgement calls on eligible patients who would likely benefit the most. The automatically generated call list, that was described as helpful by technicians, also caused unnecessary calls to unwilling patients. Promotional materials, such as flyers, were not always reported to be in use, but were always reported to be helpful by technicians when they did have them. The most frequent goal for enrollment of new patients within the program was stated to be two patients per week. Two pharmacies had no goal for patient enrollments and one pharmacy had a goal of 5–10 enrollments/week.

## 4. Discussion

This qualitative study explored pharmacy technician perspectives of their role in ABM and found that they felt the program increases adherence and convenience for patients, but there were hurdles to effective implementation remaining. Establishing the role of the technician in ABMS services is critical as pharmacist-extender involvement in clinical service delivery is associated with a service implementation success, for frequent and diverse clinical service offerings, and improved quality of work life for both pharmacists and pharmacy technicians [17,18,19,20,21,22,23,24]. However, integration of technicians into advanced or clinical support roles can prove challenging [21,24,25,26]. Understanding their unique perspectives on these new roles can be instrumental in designing training programs and assisting pharmacy leadership in the selection of technicians for these advanced roles [27].

Some important takeaways were uncovered in this exploratory study related to technician work life. The semi-structured interviews found that technicians felt their help to patients was appreciated. This team member buy-in of the ABM process is key for pharmacists to leverage the workflow efficiencies generated to enable clinical interventions such as CMR [17]. Downstream, this may have important implications for technicians’ perceived quality of work life (QOWL), which has previously reported to be low among pharmacy technicians practicing in a community pharmacy [25,28,29]. Moreover, such advanced roles may decrease technician turnover and further improve QOWL, as lack of career advancement is a known QOWL issue among pharmacy technicians [28].

Evaluation of the content within the Adaptability and Relative Priority constructs found that siloed prescriptions in the ABM portal could be a source of time-consuming erroneous fills. Housing the platform used for ABM within the pharmacy management system or enabling dynamic profile updating is necessary. The ABM interface must reflect the most current version of the patient profile to ensure that erroneous fills do not occur. This is a patient care concern that reduces pharmacy team support. Also, the report that too little time was budgeted to implement the program and complete other duties that must take priority worsened technician perceptions of the program. To prevent this, it is critical that new projects are integrated into existing workflows appropriately through accurate modeling of the workforce hours required. 

The Champion construct emerged during the interviews as a successful aspect of implementation. Pharmacy technicians who recognized the importance of the program and took initiative to implement it were associated with success. By tailoring each ABM encounter to the specific needs of the patient, these champions generated more process buy-in from patients and team members. This generated synergy and reduced unnecessary prescription fills. Interviews found the champions were well-informed about the intervention process and felt comfortable integrating it into workflow. Our study indicates that empowering technicians on the local level to take initiative when patients are struggling with a high volume of prescriptions and poor adherence is an effective approach to circumventing the inefficiencies around the automated enrollment processes.

The variability in training reported by technicians is another barrier to implementation identified. Some team members felt that although they had a great explanation of ABM conceptually, they had little exposure to the actual interface. Training specific to the ABM user interface is needed to ensure the success of the program. Hands-on training will reduce the likelihood of erroneous fills and wasted time in the enrollment process. 

Significant improvements to the ABM program may be made to better patient care and increase efficiency in the business operations of pharmacies that implement it. Effective implementation of medication synchronization core components within ABM is key to ensure that this program is effective. These components include the identification and enrollment of patients, inclusion of a medication review and patient assessment, the alignment of refills, a formal process for preparation of medications, and the delivery of medications and other services [7,30]. There is support in the literature for implementing a vaccination assessment program with ABM also. The CMR component of ABM has been proven to detect medication errors and successfully promote vaccination for patients [31]. The patients who benefit most are older adult patients with multiple medications, or have a chronic condition such as diabetes, COPD, or asthma [32]. The medical complications associated with medication nonadherence are worsening. Without proper management, nonadherence will continue to increase hospitalizations and raise costs in the health care system. This study contributes important data on pharmacy technicians, an understudied stakeholder whose perspectives are key to effectively implement patient care initiatives in a community pharmacy [7,30].

### Study Limitations

Analysis of the relative success of each program was limited due to the lack of enrollment statistics for each pharmacy and direct indicators of patient opinion. This study was limited to supermarket chain pharmacies and may not have generalizability to all other pharmacies in the community setting. A small number of interviews occurred due to saturation of themes which may limit external validity of the study, as technicians were from only one grocery store chain representing a small geographical area. These qualitative results may also have limited generalizability to other countries, due to ABM not being implemented in the community pharmacy setting. However, the study’s findings provide a helpful understanding of affective factors that are important to consider in the pre-implementation phase for any service implemented in the community pharmacy setting. Expanding this research to personnel in multiple settings may be beneficial and is warranted to discover new themes. The information technology (IT) issues specific to this supermarket chain may limit generalizability to some extent. However, most chain pharmacies contract with third party vendors to automate the enrollment process, so IT issues may persist.

## 5. Conclusions

Results from this study signify that ABM implementation can be challenging. Better ABM portal integration with the pharmacy patient profile, the promotion of champions, and appropriate workforce budgeting are key to the continued success for the program.

## Figures and Tables

**Table 1:** Interview guide.

**Section 1: Participant Demographics**
1. Are you a certified technician?
2. How long have you been working as a technician?
• [PROBE] How long have you been working at this store? • [PROBE] How long have you been working for Kroger?
3. On average, how many hours do you work per week?
4. Describe your roles and responsibilities in executing the medication synchronization program at your store.
**Section 2: Adaptability**
5. What changes, if any, would you like to make to the program?
**Section 3: Patient Needs and Resources**
6. What is your perception of patient satisfaction with the medication synchronization program?
7. What are the benefits, if any, of medication synchronization to the pharmacy?
**Section 4: Access to Knowledge and Information**
8. Explain the training, if any, that you received to carry out the roles and responsibilities you described above. • [PROBE] Do you feel the training prepared you to carry out the roles and responsibilities expected of you? • [PROBE] Why or why not?
**Section 5: Relative Priority**
9. Walk me through your process for adding medication synchronization into your workflow. • [PROBE] Do you feel that you have adequate time to implement this program?
10. What are some of the barriers, if any, your pharmacy has faced when implementing medication synchronization? • [PROBE] How has your pharmacy worked to overcome these barriers?
**Section 6: Champion**
11. Does your store have a designated champion for the medication synchronization program?
**Section 7: Intervention Participants**
12. Tell me an example of how you are informing patients about this program? • [PROBE] What promotional materials, if any, do you use to communicate the availability of this program? • [PROBE] How do you select which patients to promote this program to?

**Table 2:** Interview constructs, definitions, and illustrative quotations.

Construct	Definition	Illustrative Quotation
Access to Knowledge and Information	Ease of access to digestible information and knowledge about the innovation and how to incorporate it into work tasks.	“Yeah, I mean we went, but that’s no good until you’re in Med Sync, it doesn’t explain it in any way. It’s useless.”
Relative Priority	Individuals’ shared perception of the importance of the implementation within the organization.	“With the recent cuts and hours, no we don’t. Because the 30 minute enrollment visits take away the pharmacists and we used to have two pharmacists at all times, but now some days we only have one. To take the pharmacist out of the work flow is very detrimental to the actual work flow.” “...they have us with so many things...I am trying to get the MedSync people 90 days at the same time, make one phone call and do all my phone calls. I don’t know if that’s right or wrong.”
Patient Needs and Resources	The extent to which the needs of those served by the organization (e.g., patients), as well as barriers and facilitators to meet those needs, are accurately known and prioritized by the organization.	“I think more middle aged and younger people are satisfied with the program, but the elderly seem to get very confused, and don’t understand.”
Adaptability	The degree to which an innovation can be adapted, tailored, refined, or reinvented to meet local needs.	“I make sure I get a list of the medications they do want to be included on Med Sync and try not to mark the ones that they don’t want because that saves a lot of time, and money, and headache for the patients when they come in.” “Since our patients are older here, their medicines change all the time. They get here and they pick up medicines and it’s not what they want. Then they wanna bring it back but our policy is you can’t bring it back, therefore they get a little mad at us.”
Champions	“Individuals who dedicate themselves to supporting, marketing, and ‘driving through’ an [implementation]”, overcoming indifference or resistance that the innovation may provoke in an organization.	“I kind of pay attention to patients who are voices their concerns on making multiple trips, and also patients who have five or more medications that are falling on different dates. I go back and look at their profile and then I’ll say, “Oh, hey, we’ve got this new program called Med Sync where we can synchronize your prescription. That way, you’ll make one trip, get all your prescriptions right then," and most of the time we can get 90-day supplies, which really saves a lot of time, and in some cases, it saves a lot of money.”
Innovation Participants	Individuals served by the organization that participate in the innovation, e.g., patients in a prevention program in a hospital.	“We tell people, we have some little flyers that we pass out, and we have people, anybody that seems to benefit from it, that if they normally have a caregiver that picks up for them and they don’t get out, so it’s not as convenient for them to make five trips a month… So we’re just talking to the patients, and I have called several people that are on the recommended list and some people were not very receptive to that. She told me I was trying to get all of her money, so we did not, we deleted her. We have had a list of insurances that will pay and work with this, and so we’ve pulled patients from that, patients we know could benefit from the service.”

## References

[1] Chronic Disease Overview. CDC.gov. https://www.cdc.gov/chronicdisease/index.htm.

[2] (2003). Chapter II: The magnitude of the problem of poor adherence. In: World Health Organization. http://www.who.int/chp/knowledge/publications/adherence_full_report.pdf..

[3] Neiman A.B., Ruppar T., Ho M., Garber L., Weidle P.J., Hong Y., George M.G., Thorpe P.G. (2017). CDC Grand Rounds: Improving Medication Adherence for Chronic Disease Management — Innovations and Opportunities. MMWR. Morb. Mortal. Wkly. Rep..

[4] Krumme A.A., Glynn R.J., Schneeweiss S., Gagne J.J., Dougherty J.S., Brill G., Choudhry N.K. (2018). Medication Synchronization Programs Improve Adherence To Cardiovascular Medications And Health Care Use. Heal. Aff..

[5] Bernard K., Cowles B., McCall K., Henningsen R.M., O’Toole M., Tu C. (2019). Impact of medication synchronization programs on proportion of days covered (PDC) scores and Medicare Part D medication-related adherence metrics. J. Am. Pharm. Assoc..

[6] Chater R.W. Improving Quality Care: The Appointment-Based Model. https://www.pharmacytimes.com/publications/directions-in-pharmacy/2015/march2015/improving-quality-care-the-appointment-based-model.

[7] Patti M., Renfro C., Posey R., Wu G., Turner K., Ferreri S.P. (2019). Systematic review of medication synchronization in community pharmacy practice. Res. Soc. Adm. Pharm..

[8] Watson L.L., Bluml B.M. (2013). Pharmacy’s Appointment Based Model Implementation Guide for Pharmacy Practice Distributed in partnership with APhA and NASPA. American Pharmacists Association Foundation Website. www.aphafoundation.org.

[9] Bonner L. (2015). Med Sync Catching on across Nation. American Pharmacists Association Website. https://www.pharmacist.com/article/med-sync-catching-across-nation?is_sso_called=1.

[10] Consolidated Framework for Implementation Research (CFIR): Codebook Template. https://cfirguide.org/constructs/.

[11] Lewis S.B., Price H.K., Stafford R.A. (2008). The Effect of Appointment-Based Medication Synchronization on Clinical Services in a Community Pharmacy. Pharmacy.

[12] Holdford D. Simplify My Meds® Appointment-based Medication Synchronization Pilot Study Report: Prepared for National Community Pharmacists Association. http://www.ncpa.co/pdf/ncpa-ABM-report.pdf.

[13] Patterson J.A., Holdford D.A., Saxena K. (2016). Cost-benefit of appointment-based medication synchronization in community pharmacies. Am. J. Manag. care.

[14] Ulin P.R., Robinson E.T., Tolley E.E. (2005). Qualitative Methods in Public Health: A Field Guide for Applied Research. Med. Sci. Sports Exerc..

[15] Tong A., Sainsbury P., Craig J. (2007). Consolidated criteria for reporting qualitative research (COREQ): A 32-item checklist for interviews and focus groups. Int. J. Qual. Heal. Care.

[16] Guest G., Bunce A., Johnson L. (2006). How Many Interviews Are Enough?. Field Methods.

[17] Gernant S.A., Nguyen M.-O., Siddiqui S., Schneller M. (2017). Use of pharmacy technicians in elements of medication therapy management delivery: A systematic review. Res. Soc. Adm. Pharm..

[18] Mattingly A., Mattingly T.J. (2018). Advancing the role of the pharmacy technician: A systematic review. J. Am. Pharm. Assoc..

[19] Hohmeier K.C., Garst A., Adkins L., Yu X., Desselle S.P., Cost M. (2019). The Optimizing Care Model: A novel community pharmacy approach to enhance patient care delivery by leveraging the technician workforce through technician product verification. J. Am. Pharm. Assoc..

[20] Hohmeier K.C., McDonough S.L., Rein L.J., Brookhart A.L., Gibson M.L., Powers M.F. (2019). Exploring the expanded role of the pharmacy technician in medication therapy management service implementation in the community pharmacy. J. Am. Pharm. Assoc..

[21] Hohmeier K.C., Desselle S.P. (2019). Exploring the implementation of a novel optimizing care model in the community pharmacy setting. J. Am. Pharm. Assoc..

[22] Lengel M., Kuhn C.H., Worley M., Wehr A.M., McAuley J.W. (2018). Pharmacy technician involvement in community pharmacy medication therapy management. J. Am. Pharm. Assoc..

[23] Bright D., Klepser M.E., Murry L., Klepser D.G. (2018). Pharmacist-Provided Pharmacogenetic Point-of-Care Testing Consultation Service: A Time and Motion Study. J. Pharm. Technol..

[24] Justis L., Crain J., Marchetti M.L., Hohmeier K.C. (2016). The Effect of Community Pharmacy Technicians on Industry Standard Adherence Performance Measures After Cognitive Pharmaceutical Services Training. J. Pharm. Technol..

[25] Desselle S.P. (2005). Job Turnover Intentions Among Certified Pharmacy Technicians. J. Am. Pharm. Assoc..

[26] Desselle S.P., Holmes E.R. (2007). Structural model of certified pharmacy technicians’ job satisfaction. J. Am. Pharm. Assoc..

[27] Moya A., Unni E., Montuoro J., Desselle S.P. (2019). Engaging pharmacy technicians for advanced clinical support tasks in community pharmacies: A cluster analysis. J. Am. Pharm. Assoc..

[28] Desselle S.P. (2005). Survey of certified pharmacy technicians in the United States: a quality-of-worklife study. J. Am. Pharm. Assoc..

[29] Desselle S.P., Holmes E.R. (2017). Results of the 2015 National Certified Pharmacy Technician Workforce Survey. Am. J. Heal. Pharm..

[30] Renfro C., Patti M., Ballou J., Ferreri S.P. (2018). Development of a medication synchronization common language for community pharmacies. J. Am. Pharm. Assoc..

[31] Ariyo O., Kinney O., Brookhart A., Nadpara P., Goode J.-V. (2019). “Kelly” R. Medication therapy problems and vaccine needs identified during initial appointment-based medication synchronization visits. J. Am. Pharm. Assoc..

[32] Luder H., Kunze N., Heaton P.C., Frede S.M. (2018). An appointment-based model to systematically assess and administer vaccinations. J. Am. Pharm. Assoc..

